# Profile of Neurosyphilis Patients Admitted to a Tertiary Care Centre of Nepal: An Observational Study

**DOI:** 10.31729/jnma.8981

**Published:** 2025-05-31

**Authors:** Uma Giri, Laila Lama Tangbetani, Anupama Karki, Jagat Jeevan Ghimire

**Affiliations:** 1 National Academy of Medical Sciences, Bir Hospital, Mahaboudha, Kathmandu, Nepal; 2 Kanti Children Hospital, Maharajgunj, Kathmandu, Nepal

**Keywords:** *cerebrospinal fluid; neurosyphilis*, *profile*, *treatment*

## Abstract

**Introduction:**

Neurosyphilis is a rare complication of untreated syphilis with limited literature. We aimed to look for demographic characters and clinical presentation of patients admitted with the diagnosis of neurosyphilis.

**Methods:**

This was an observational cross-sectional study that included analysis of records of neurosyphilis patients admitted to the National Academy of Health Sciences, Kathmandu, Nepal, from May 2015 to April 2024. All patients diagnosed with neurosyphilis were included, while those with incomplete data were excluded. The Centers for Disease Control and Prevention criteria were used to define and categorize neurosyphilis. Continuous variables were summarized as mean ± standard deviation for normally distributed data or as median and interquartile range for skewed data. Categorical variables were described using numbers and percentages.

**Results:**

A total of 53 cases were included in the study, with 31 (58.49%) males. The median age was 46 years (interquartile range: 37-60 years). Decreased vision was ovserved in 30 (56.60%) patients, and redness of the eye in 11 (20.75%) and headache in 4 (7.55%). Eye signs were observed in 43 (81.13%) patients. Cerebrospinal fluid Venereal Disease Research Laboratory (VDRL) test was positive in 11 (20.75%) cases. Based on Centers for Disease Control and Prevention criteria, 11 (20.75%) patients had verified neurosyphilis, 34 (64.15%) had likely neurosyphilis, and 8 (15.09%) had possible neurosyphilis. Iintramuscular benzathine penicillin was administered in 26 (49.06%) patients.

**Conclusions:**

There were more male patient with Neurosyphilis in our population. Decreased vision was the most common presentation. Likely neurosyphilis was most common diagnosis among studied population.

## INTRODUCTION

Syphilis is caused by Treponema pallidum with an annual incidence of 8 million cases in adults aged 15-49 years.^1^ Around 16-48% of patients with entry of Treponema Pallidum into Central nervous system develop abnormalities of cerebrospinal fluid(CSF).^2,3^ Although majority of them have spontaneous resolution, minority of them persist in the form of asymptomatic or symptomatic neurosyphilis with estimated incidence from 0.47 to 2.1 cases per 100000 population.^3-5^

Diagnosis of neurosyphilis is challenging as neurosyphilis can affect every part of Central nervous system and mimic a wide variety of neurological and mental disorders. Cerebrospinal fluid analysis for nontreponemal tests as veneral disease research laboratory (VDRL) and analysis for protein, cells is a crucial diagnostic test for the diagnosis of neurosyphilis.^6^ There have been an increase in number of neurosyphilis cases in Human immunodeficiency virus era. ^7-10^

In view of limited literature data on neurosyphilis from Nepal, we conducted this study with the objectives of looking into demographic characters, clinical presentation and treatment of patients admitted with the diagnosis of neurosyphilis.

## METHODS

This was an observational cross-section study that include analysis of the record of neurosyphilis patients admitted to National Academy of Health Sciences, Kathmandu, Nepal from May 2015 to April 2024. Ethical approval was taken from Institutional review board (Reference number: 813). All patients with the diagnosis of neurosyphilis were included in the study. Those patients who had incomplete data were excluded from the study. Definition of Centre for disease control (CDC) was used to define and categorize neurosyphilis.^11^

Possible Neurosyphilis was defined as infection of the central nervous system with Treponema pallidum, as evidenced by manifestations including syphilitic meningitis, meningovascular syphilis, general paresis, including dementia, and tabes dorsalis. Likely neurosyphilis was defined a case of possible neurosyphilis with elevated cerebrospinal fluid (CSF) protein (>50 mg/dL) or leukocyte count (>5 white blood cells/cubic millimeter in the absence of other known causes of these abnormalities. Verified neurosyphilis was defined as a case of probable neurosyphilis with reactive Veneral Disease Research Laboratory(VDRL) in Cerebro spinal Fluid (CSF) in the absence of grossly bloody contamination.

In this retrospective study, medical record of each patient was extensively reviewed for demographic, clinical and laboratory data. Records of patients meeting the inclusion criteria were entered in predefined proforma. Data on demography as age, gender, level of education, symptoms at presentation, clinical signs were recorded along with laboratory findings such as serum Veneral Disease Research Laboratory (VDRL), Treponema pallidium hemagglutination assay (TPHA), Cerebro spinal fluid (CSF) cells, protein and CSF veneral disease research laboratory(VDRL).

Data were entered on Excel and analyzed in SPSS Statistics for Windows version 22 (SPSS Inc., Chicago, III., USA) . For variables with continuous data, the normal distribution was described as mean ± standard deviation, and the skewed distribution was described as median (M) and quartile range (IQR). The categorical variables were described by numbers and percentages (%).

## RESULTS

A total of 56 medical charts were identified during the review of medical records. Three (5.36%) charts were excluded due to incomplete data, and data from 53 patients were analyzed. There were 31 (58.49%) males and 22 (41.51%) females. The median age of the study population was 46 years (IQR range: 37-60 years), (Table 1).

A total of 30 (56.60%) patients presented with decreased vision, followed by redness of the eyes. Ocular presentation was observed in 43 (81.13%) patients. Uveitis was present in 44 (83.02%) cases, followed by loss of accommodation reflex and retinitis, 3 (5.66%) patients were asymptomatic (Table 2).

**Table 1:** Baseline characteristics of neurosyphillis patients admitted to tertiary care centre of Nepal (n=53).

Variables	n (%)
Gender	
Male	31 (58.49)
Female	22 (41.51)
Education level	
Secondary school	1 (1.96)
High school	4 (7.84)
Bachelor	1 (1.96)
Masters	1 (1.96)
Literate	8 (15.69)
Illiterate	6 (11.76)
Not available	32 (60.41)
Risky sexual behaviour	
Yes	41 (77.41)
No	12 (22.59)

**Table 2:** Clinical presentation ofneurosyphillis patients admitted to tertiary care centre of Nepal (n=53).

Symptom	n (%)
Decreased vision	30 (56.60)
Redness of eyes	11 (20.75)
Headache	4 (7.55)
Disturbance of gait	3 (5.66)
Photophobia	1 (1.89)
Seizure	1 (1.89)
Asymptomatic	3 (5.66)
Signs	
Ocular signs	43 (81.13)
Uveitis	44 (83.02)
Retinitis	1 (1.89)
Scleritis	1 (1.89)
Loss of accommodation	2 (3.77)
Pupillary reflex abnormalities	3 (5.66)
Positive Romberg sign	3 (5.66)
No sign	4 (7.66)

**Table 3:** Laboratory findings of neurosyphillis patients admitted to tertiary care centre of Nepal (n=53).

Serum	
Serum VDRL reactive n(%)	53 (100)
Blood TPHA positivity	53 (100)
Serum VDRL titer	
1:2	19 (35.85)
1:4	15 (28.30)
1:8	4 (7.55)
1:16	7 (13.21)
1:32	8 (15.09)
HIV	
Positive	5 (9.40)
Negative	48 (90.60)
CSF	
CSF VDRLReactive	11 (20.75)
CSF cells (>5 cells)	26 (49.06)
CSF(>50 mg/dl) protein	40 (75.47)
CSF titre	
1:2	1 (9.09)
1:4	1 (9.09)
1:8	6 (54.55)
1:16	1 (9.09)
1:32	2 (18.18)

**Table 4:** Treatment of neurosyphilis patients admitted to tertiary care centre of Nepal (n=53).

	PN n (%)	LN n (%)	VN n (%)	TN n (%)
A	8 (100)	18 (52.94)	0 (0.00)	26 (49.06)
B	0 (0.00)	15 (44.34)	9 (81.82)	24 (45.28)
C	0 (0.00)	0 (0.00)	2 (18.18)	2 (3.77)
D	0 (0.00)	1(2.94)	0 (0.00)	1 (2.94)

PN=Possibleneurosyphilis;LN=Likely neurosyphilis, VN=Verified neurosyphilis; TN=Total neurosyphilis; A= Inj. Benzathine penicillin; B= Inj. Benzyl penicillin;C= Inj. Ceftriaxone; D=Tab. Erythromycin

The serum VDRL was reactive in all 53 (100%) cases. The serum VDRL titer was 1:2 in 19 (35.85%) patients, followed by 1:4 in 15 (28.30%) patients. A total of 5 (9.43%) neurosyphilis patients were seropositive for HIV. All HIV-positive patients were male, with a mean age of 41 years. Each HIV-positive patient presented with decreased vision and uveitis. Among the 4 HIV-positive patients, both protein and cell counts were elevated above 50 mg/dL and 5 lymphocytes per microliter, respectively. Cerebrospinal fluid (CSF) VDRL was positive in 11 (20.75%) patients. Among CSF VDRL reactive cases, 6 (54.55%) had a 1:8 titer, and 2 (18.18%) had a 1:32 titer. CSF protein was elevated in 40 (75.47%) patients, and CSF cells were elevated in 26 (49.06%), (Table 3). There were 8 (15.09%) possible neurosyphilis, 34 (64.15%) likely neurosyphilis and 11(20.75%) verified neurosyphilis.

The treatment was given on the basis of diagnosis and availability of medicine. A total of 26 (49.06%) neurosyphilis patients were treated with injection benzathine penicillin. injection benzyl penicillin was used for treating 24 (45.28%) patients, and injection ceftriaxone for 2 (3.77%) patients. One (1.89%) patient who had penicillin allergy was treated with tablet erythromycin and was kept on regular follow up (Table 4).

## DISCUSSION

Neurosyphilis usually presents in 40s. This has been the observations from various studies by Zhang et al, Daey Ouwens et al and Drago et al.^5,12,13^ Our study showed similarities to these studies with the median age of 46 years. In contrast to our report, studies from San Fransisco by Flood et al, Timmersmans et al from South Africa showed the age at presentation in lower age group with the median age of 39 years (22-88years) and 39 years (17-67 years) respectively.^8,14^ The difference may have been due to the fact that these cohort included subjects with HIV positivity, where the age of presentation of neurosyphilis is at younger age compared to HIV negative. In our study number of subjects with HIV positivity was very low.

In our study proportion of males were higher than that of females with 58.49% of cases being male. However, it is not possible to compare the occurance between male and female as this study has not compared these prevalences. Nevertheless, in various studies done by Flood et al, Daey Ouwens et al, Drago et al and Zhang et al the proportion of female subjects were as low as 9 % and as high as 29%.^8,5,12,13^ Report of our study differed from the study by Daey Ouwens et al from Netherland where there was female predominance of cases. Social issues making the less likelihood of hospital visit may be the reason for lower female proportion in our study.

Syphilis increases the risk of HIV acquisition by three folds. Syphilis can exacerbate HIV infection and inflammation in the central nervous system, increasing HIV viral load and decreasing CD+T lymphocytes count. Syphilitic lesion may also provide access to HIV acquisition. Patients with neurosyphilis may have HIV seropositivity posing a great diagnostic and therapeutic challenges to clinicians.^15^ In a clinical cohort of HIV infected patients by Ghanem et al neurosyphilis was common in male (81%) with median age 40 (26-68), 66% neurosyphilis cases were symptomatic with the most common sign being uveitis.^9^ In our study HIV seropositivity was in 5 (9.40%) subjects. This result was similar to the study by Drago et al where HIV seropositivity was noted in 29 (10%) patients.^13^ In our study among HIV positive patients all were male with mean age 41 years. All HIV positive patients presented with decreased vision and uveitis. In 4 HIV positive patients, both protein and cells were raised above 50mg/dl and 5 lymphocytes respectively. Serum VDRL titre was 1:32 in only one HIV positive case and 3 of them had verified syphilis with positive CSF VDRL. In contrasts to our study Flood et al^8^ showed 64% neurosyphilis patients had HIV positivity with male predominance, median age 39, high serum VDRL titre (median, 1:128 at presentation), 33% had early neurosyphilis and 5% had late neurosyphilis manifestations. The difference might be due to the fact that our patients were predominantly referred from ophthalmology unit in contrast to other study and also the study by Flood et al was conducted during HIV era.

Syphilis has a variable presentation. In pre antibiotic era tabes dorsalis and general paresis of insane were the hallmark presentation of neurosyphilis. The clinical manifestation of neurosyphilis rapidly changes with the rampant use of antibiotics. Common presentation of neurosyphilis include ocular symptoms, neuropsychiatric and neural presentation. The study by Mitsonis et al^16^ showed that typical symptoms of neurosyphilis like tabetic symptoms, VIII cranial nerve involvement and headache decreased in the year 1985 to 2005 however, 85.70% had atypical symptoms like cognitive impairment and mental disorder. One third of the studied population had tabes dorsalis in a study by Ouwens et al.^5^ In Zhang et al study the major presentation was neuropsychiatric event.^12^ On the contrary, in our study most of the patients had ocular presentation and they were referred from the department of ophthalmology for evaluation. Decreased vision was the most common presentation in our series. It was followed by redness of eyes and loss of accommodation reflex. The difference in presentation with the studies probably resulted from the fact that in these cohort patients were referred from a neuropsychiatric unit in contrast to our study where patients were referred from ophthalmology department.

Treponema pallidum can infect any ocular tissue in all stages of syphilis resulting ocular syphilis. Uveitis and optic neuropathy are the commonest ocular manifestations. Ocular syphilis presenting as neurosyphilis is defined when the cases meet the clinical definition of neurosyphilis in the form of elevated CSF protein or CSF cells and/or CSF VDRL. Ocular syphilis is the manifestation of early neurosyphilis.^17^ In a cohort of 10 subjects of uveitis associated with neurosyphilis the most common presentation was vision loss.^18^ Although ocular syphilis is one of the manifestations of primary syphilis and secondary as well, it's now a very noticed manifestation of neurosyphilis and there are cohort explaining the ocular manifestation in the form of redness of eyes and dimness of vision.^19^ The current study also showed similar trend in the form of predominant ocular presentation.

The diagnosis of neurosyphilis is based on serum and CSF VDRL. For patients with ocular and neurologic symptoms or serofast status, lumbar puncture is done to evaluate CSF protein, sugar, CSF cells and CSF VDRL. Although CSF VDRL is regarded as the gold standard for the diagnosis of neurosyphilis, its specificity is high (89-96%) but the sensitivity is relatively low (12-48%).^20-22^ We also used similar technique for the diagnosis of neurosyphilis in our setting and based on the pattern of positivity of CSF VDRL, CSF protein, CSF cells and clinical features neurosyphilis is classified as possible neurosyphilis, likely neurosyphilis and verified neurosyphilis.^11^ In our study, there were 8 (15.09%) possible neurosyphilis, 34 (64.15%) likely neurosyphilis and 11 (20.75%) verified neurosyphilis. Hence, the cases of verified neurosyphilis was low in our study which was comparable to published series.^23^

In our study serum VDRL and serum TPHA was reactive in all patients. The previous study showed that high serum titre had more likely chance of having neurosyphilis. Serum VDRL of 1:32 titre had been suggested as a cutoff for doing lumbar puncture suspecting neurosyphilis irrespective of HIV positivity.^24^ In our study 19 (35.85%) had 1:2 serum VDRL titre and 15 (28.30%) had 1:4, hence 64.15% were having low titre.19 (35.85%) patients had 1:8 and more titre. Hence, our study does not support serum VDRL titre >1:32 as a cut off point for doing lumbar puncture. CSF VDRL was reactive in 11 (20.75%). The CSF cell analysis showed more than 5 in 26 (49.06%) patients. The CSF protein was more than 50mg/ dl in 40 (75.47%) patients. These laboratory findings were comparable to other studies.^25^

Benzyl Penicillin has been the drug of choice for the management of neurosyphilis both in immunocompromised and immunocompetent patients. Ceftriaxone has been shown to be alternative to penicillin in the management of neurosyphilis.^26^ There is a lack of strong evidence on the selection of drug in the management of neurosyphilis. There are small cohort studies that showed variable cure rate of 44 to 82% in the ceftriaxone group and 18-82% in penicillin group respectively. Apart from penicillin tetracycline, erythromycin and chloramphenicol have been used to treat neurosyphilis.^27^ In our study, Benzyl penicillin was used to treat neurosyphilis in 45.3% of case followed by ceftriaxone in 3.8% of cases. Our study showed similarities to various studies. However, we did not have data on cure rate of the study population, hence we could not discuss on the cure rate of penicillin versus ceftriaxone in our study.

This study adds data on the pattern of presentation of neurosyphilis in HIV era from a tertiary care center from Kathmandu, Nepal. This can help in understanding the demography and clinical presentation of patients of neurosyphilis. Also, the study highlights that ocular symptom in a patient with syphilis is an alert sign and it should be evaluated with an immense care. However, this was a retrospective study from a single center and a full detailed history on risky sexual behavior, previous syphilis and use of drugs in HIV could not be extracted. Sample size was also small. Multicentric study with adequate data on risk factors, treatment given and outcome can help understand the epidemiology of the disease in our context.

## CONCLUSION

The Ocular presentation was the most common presentation in our study and redness of eyes was the most common symptoms. Likely neurosyphilis as defined by clinical features, elevated protein and /or sugar was the most common diagnosis of the studied population. Serum VDRL titre of >1:32 may not be the indication for lumbar puncture in HIV negative patients.
